# Characterization of a Dengue Virus Serotype 1 Isolated from a Patient in Ciudad Juarez, Mexico

**DOI:** 10.3390/pathogens10070872

**Published:** 2021-07-10

**Authors:** Pedro M. Palermo, Antonio de la Mora-Covarrubias, Jeanette Orbegozo, Jessica A. Plante, Kenneth S. Plante, Florinda Jimenez-Vega, Douglas M. Watts

**Affiliations:** 1 Department of Biological Sciences and Border Biomedical Research Center, University of Texas at El Paso, El Paso, TX 79968, USA; jporbegozor@miners.utep.edu (J.O.); dwatts2@utep.edu (D.M.W.); 2 Instituto de Ciencias Biomedicas, Universidad Autonoma de Ciudad Juarez, Juarez City 32100, Mexico; adelamor@uacj.mx (A.d.l.M.-C.); fjimenez@uacj.mx (F.J.-V.); 3 Department of Microbiology and Immunology, University of Texas Medical Branch, Galveston, TX 77555, USA; japlante@utmb.edu (J.A.P.); ksplante@utmb.edu (K.S.P.)

**Keywords:** dengue virus, Mexico, Central America clade

## Abstract

Dengue (DEN) is the most important human arboviral disease worldwide. Sporadic outbreaks of DEN have been reported since 1980 in urban communities located along the border in southeast Texas and northern Mexico. Other than the Rio Grande Valley region of TX, autochthonous transmission of DENV has not been reported from any other US border communities. As part of a surveillance program for arthropod-borne viruses in Ciudad Juarez, Mexico, during November 2015, a blood sample was obtained from a female patient who experienced an undifferentiated fever and arthralgia. The plasma of the sample was tested for virus in Vero-76 and C6/36 cells. DENV serotype 1 (DENV-1) was isolated in the C6/36 cells, and nucleotide sequencing of the envelope gene and full genome grouped the DENV-1 isolate in the Central America clade. The patient had not traveled outside of Ciudad Juarez, Mexico, thus suggesting DENV-1 infection was acquired in this community.

## 1. Introduction

Dengue is the most important mosquito-borne viral human disease in the tropical and subtropical regions of the world. The dengue disease is caused by any of the four (1–4) dengue virus (DENV) serotypes, which can range from a mild febrile illness to severe hemorrhagic fever and/or fatal shock syndrome. DENV is transmitted by the *Aedes aegypti* and *Ae. albopictus* mosquitoes, and the distribution of both species has increased in the last 30 years, mainly in the temperate regions of Europe and North America [1,2].

Dengue cases in the Americas have increased over time from 1980 to 2007, with Mexico being the country with the third highest number of dengue cases [3]. *Ae. aegypti* is widely distributed in Mexico, including the urban communities in the southern, western, and eastern regions, extending along the Gulf Coast as far north as Matamoros, Mexico, as well as the southern half of the United States (US) [2,4]. Along the US–Mexico border, DENV is endemic only in the southern region of Texas, even though *Ae. aegypti* is found in almost all urban border communities. More recently, data accumulated suggested that DENV is endemic in Ciudad Juarez, Mexico, an urban community in the state of Chihuahua located in Northwestern Mexico, which is the second-largest community located along the US–Mexico border. The first reported evidence was the detection of DENV ribonucleic acid (RNA) in *Ae. aegypti* mosquitoes in Ciudad Juarez [5]. Subsequently, serological evidence of DENV infection in humans was also reported in the Anapra neighborhood of Ciudad Juarez, including seroconversions to DENV infection in 10.4% (*n* = 5) of 48 individuals who were negative for DENV antibody during the baseline survey [6]. The individuals who seroconverted did not report any travel outside of the community, thus suggesting that infection was acquired as a result of local DENV transmission. Further evidence suggesting that DENV is endemic in the Juarez community was supported by the observation reported in this study of the isolation of DENV-1 in November 2015 from a febrile patient in the Felipe Angeles neighborhood located adjacent to the Anapra community of Ciudad Juarez, Mexico. 

## 2. Results

**Figure 1:**
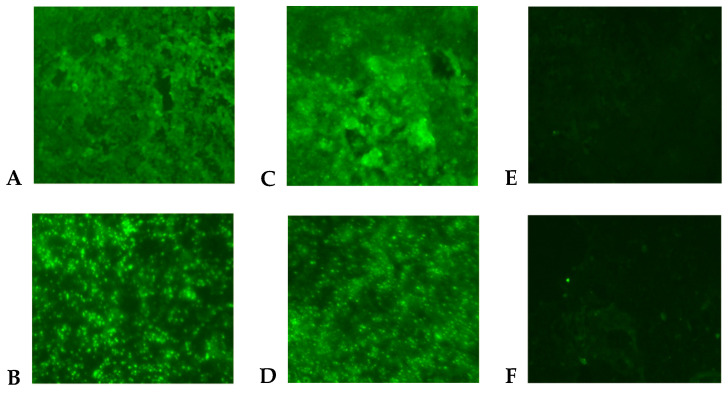
Indirect immunofluorescent antibody assay in C6/36 cells (200× magnification). Cells were inoculated with DENV-1 (**A**,**B**), human plasma (**C**,**D**), and cell culture medium (**E**,**F**). Viral antigen was detected by using polyclonal antibodies to Saint Louis encephalitis virus (**A**,**C**,**E**) and monoclonal antibody (15F3) to DENV-1 (**B**,**D**,**F**).

A *Flavivirus* was isolated from the patient’s plasma sample in the C6/36 cells according to the detection of viral antigen by immunofluorescence assay (IFA) using polyclonal Saint Louis encephalitis virus (SLEV) antibodies, which are cross-reactive to *Flavivirus* (Figure 1C). The *Flavivirus* isolate was further identified as DENV-1 using DENV-1 monoclonal antibody (Figure 1D) and by the sequencing of the generic *Flavivirus* RT-PCR amplicon (data not shown). Positive controls (DENV-1-infected C6/36 cells) were also reactive by IFA to SLEV polyclonal (Figure 1A) and DENV-1 monoclonal antibodies (Figure 1B). The infectivity titer of the DENV-1 in the plasma of the patient was 5 × 10^2^ PFU/mL at the time the blood sample was drawn.

**Figure 2:**
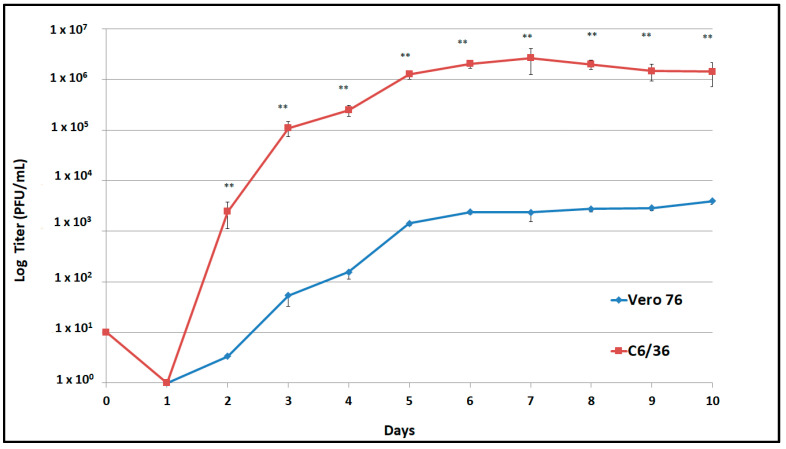
Replication curves of the Ciudad Juarez DENV-1 isolate in Vero-76 and C6/36 cells. DENV-1 isolate replicated at significant high titers (** *p* < 0.05) in C636 cells in comparison to Vero-76 cells.

Viral replication of the second passage of DENV-1 was compared in Vero and C6/36 cells up to 10 days post infection (dpi). DENV-1 replication in C6/36 cells was approximately 3 logs higher in C6/36 cells than in Vero-76 cells from 2 to 10 dpi (*p* < 0.05), reaching the highest titer (2 × 10^6^ PFU/mL) after 7 dpi (Figure 2).

**Figure 3:**
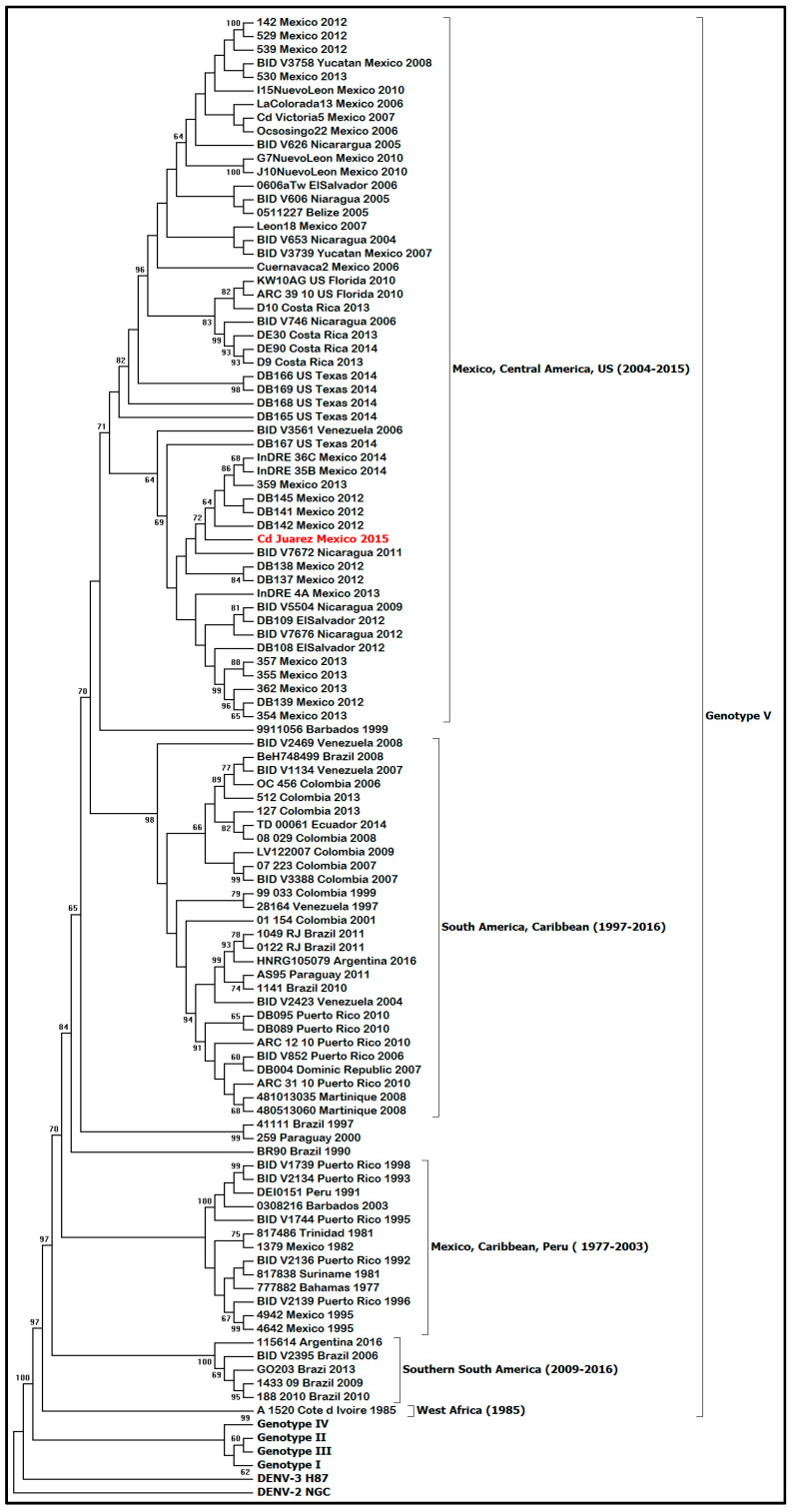
Molecular phylogenetic analysis using maximum-likelihood method derived from 100 DENV-1 envelope glycoprotein gene sequences. DENV-1 genotypes (I–V) and isolates are shown using brackets. The tree was rooted with prototype strains of DENV-3 (H87) and DENV-2 (NGC). DENV-1, strain Ciudad Juarez, is highlighted in red. The tree with the highest log likelihood (−12,372.1590) is shown.

**Figure 4:**
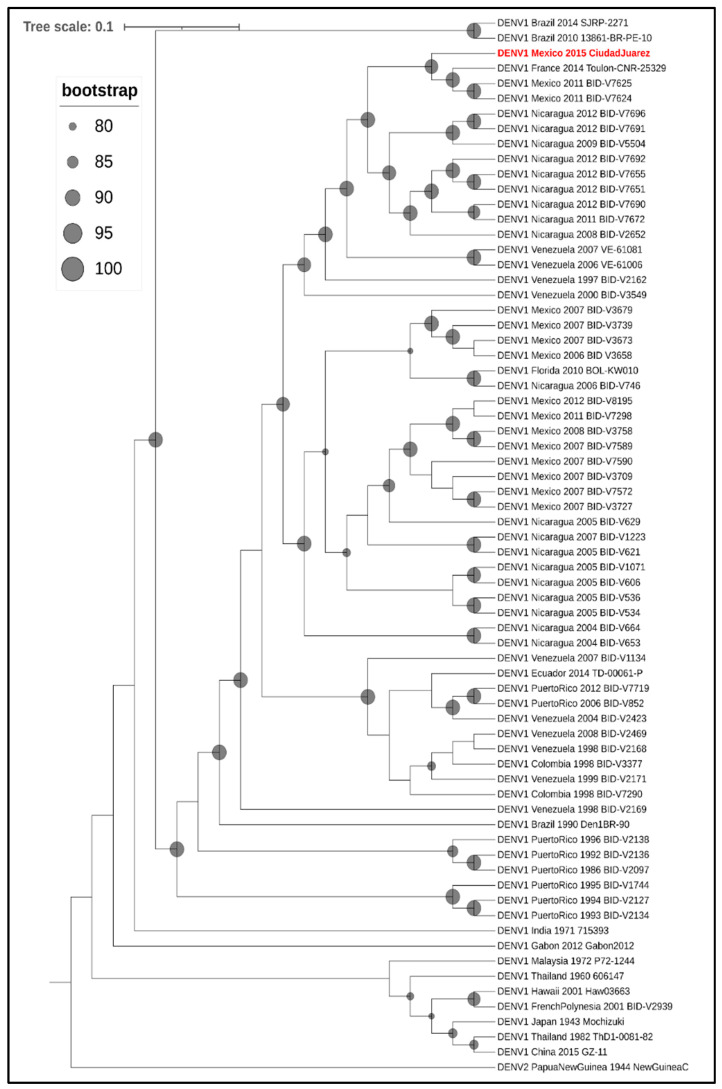
Phylogenetic analysis of DENV-1. Maximum-likelihood tree based on the full genome sequences (minus the 5′ and 3′ UTRs) of 68 strains of DENV-1 from GenBank, along with the 2015 Ciudad Juarez isolate (highlighted in red and bold text). DENV-2 strain New Guinea was included to root the tree. In the cladogram, branch lengths are ignored, but bootstrapping values ≥80 are represented as proportionately sized gray circles at the relevant nodes.

Phylogenetic analysis of the envelope gene indicated that the DENV-1 isolate clustered in genotype V and grouped with other Central American strains of DENV-1 (Figure 3) and with other DENV-1 strains isolated in Mexico (Tamaulipas, Yucatan, and Morelos) and Texas (Brownsville). Furthermore, a full genome analysis showed that the DENV-1 isolate clustered with Central American DENV-1 strains (Figure 4).

The amino-acid diversity of the DENV-1 isolate was compared with other Mexican DENV-1 strains. The DENV-1 isolate was unique amongst the other Mexican strains in the amino-acid alignment due to five amino-acid changes in the NS1 (D139E), NS2 (L41F, K218R), and NS3 (V332A, R338K) proteins (Table 1).

## 3. Discussion

The findings herein represent the first reported isolation of a DENV from a patient in Ciudad Juarez, Mexico, where previous serological evidence of DENV infection was reported among a human cohort [6]. The patient’s clinical data were consistent with a febrile illness with a DENV-1 viremia level of 5 × 10^2^ PFU/mL in the plasma and the consequent isolation of DENV-1 in C6/36 cells, in addition to being consistent with clinical criteria routinely used for DENV isolation from clinical samples [7].

Viral replication of the DENV-1 isolate was higher in C6/36 cells than in Vero cells (Figure 2); even though in vitro testing was performed, the findings suggested the ability of the DENV-1 isolate to replicate in its mosquito vector in nature. Furthermore, a limited number of passages were performed, restricting the adaptation of the DENV-1 isolate in the mosquito cell line.

Mexico has experienced several large outbreaks of dengue in the last two decades (2000–2019) causing more than 520,000 human cases [8]. Furthermore, most of the dengue cases in the last 10 years were reported from the northern states of Mexico, where DENV-1 and DENV-2 are the predominant serotypes. The DENV-1 isolate from this study clustered with other Mexican and Central American strains of DENV-1 belonging to genotype V. DENV-1 genotype V has been circulating in Mexico since the late 1970s and has experienced several lineage replacements since its introduction, causing a high degree of genetic variability [9]. Moreover, DENV-1 genotype V is widely distributed in the Americas, and its clades are associated with distinct regions (South America, Caribbean, and Central America) [10]. Furthermore, the presence of various country-specific DENV-1 founder effects may have supported the geographical structure of DENV-1 genotype V in the Americas [11].

Variation in the virus genome has been reported to be associated with virulence. For example, amino-acid substitutions on the DENV E and NS3 proteins have resulted in enhanced virulence in animal models and neurotoxicity, respectively [12,13]. Other amino-acid substitutions on the DENV NS1, NS4B, and NS5 proteins have increased the viral fitness in endemic regions [14]. Our study identified five amino-acid changes in the DENV-1 isolate that were unique in comparison with other Mexican DENV isolates (Table S1). Most of the changes involved amino acids with similar properties (e.g., NS1 (D139E), NS2 (K218R), and NS3(V332A, R338K)), which may not of have resulted in a viral protein dysfunction. Furthermore, four amino-acid changes in the Ciudad Juarez strain were also found in other DENV-1 strains, mainly from Asia. Briefly, NS1 139D was changed to N in strains from China (GIZ-11), French Polynesia (BID-V2939), Hawaii (Haw03663), Japan (Mochizuki), Malaysia (P72-1244), and Thailand (606147 and ThD1-0081-82). NS2A 41L was changed to F in Brazil (SJRP-2271), China (GIZ-11), and India (715393) strains. NS3 332V was changed to A in Brazil (SJRP-2271), Gabon (Gabon2012), and Hawaii (Haw03663), and changed to I in the Thailand (606147) strain. Lastly, NS3 338R was changed to K in the French Polynesia (BID-V2939) strain [15]. 

The results suggested that the patient acquired DENV-1 infection in Ciudad Juarez because no travel history was reported before the onset of illness. Moreover, that DENVs are endemic in this urban community is supported by the observation that three individuals seroconverted to DENV-1 infection during 2015 in Ciudad Juarez, and two of the three were positive for dengue IgM antibody [6]. Furthermore, a total of 10 human DENV-1 infections were reported in Ciudad Juarez in 2015 [16], thus documenting that the DENV-1 is endemic in this Mexican border urban community, representing a possible source for locally acquired DENV infections. A total of 12 dengue cases have been reported in Ciudad Juarez during the last 5 years, with DENV-1 being the most frequent cause of cases (*n* = 11), whereas a single case was caused by DENV-2 in 2019 [17,18,19].

An alternative explanation for our findings regarding the human cases of dengue in Ciudad Juarez is the possibility of imported dengue cases resulting in secondary focal and transient autochthonous transmission to cause one or more cases. The reported occurrence of sporadic outbreaks of dengue attributed to autochthonous transmission from 1980 to 2013 in Brownsville, Texas, and surrounding communities was associated with DENV-infected travelers returning from visits to the bordering Mexican city of Matamoros during dengue epidemics in this community [20,21,22]. Thus, a low number of secondary autochthonous cases may have been acquired from returning viremic travelers as a source of infection, before going undetected, especially in areas without active surveillance programs; furthermore, asymptomatic or silent DENV infections are more difficult to detect than symptomatic cases [23,24].

Further longitudinal cohort studies in humans and mosquito surveillance are needed to obtain a better understanding of the dynamics of DENV transmission in this Mexican border community. Lastly, the vectorial capacity of *Ae. aegypti* mosquitoes from this Mexican border region for DENV would help to provide information for predicting the risk of DENV transmission.

## 4. Materials and Methods

### 4.1. Patient and Sample Collection and Processing

**Figure 5:**
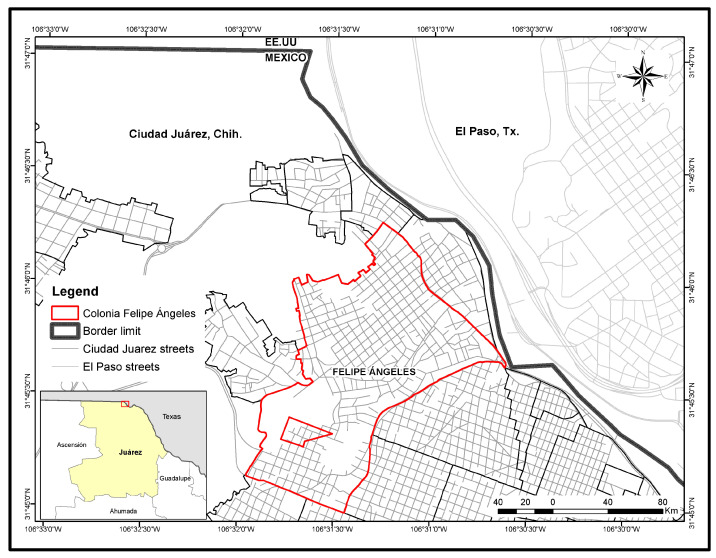
Map of Ciudad Juarez, Mexico, highlighting the adjoining neighborhood of Felipe Angeles, Ciudad Juarez, Chihuahua 32100.

A 64 year old female housewife who resided in Felipe Angeles, Ciudad Juarez, Chihuahua (Mexico) experienced a febrile illness on November 2015, which was characterized by an acute onset of chills, severe headache, fever, diarrhea, joint pain, bad taste in the mouth, and decreased appetite. She sought medical attention, and medication was administered for symptomatic treatment. A venous blood sample was collected during the acute phase of illness from the patient with a 6 mL vacutainer tube containing ethylenediaminetetraacetic acid (EDTA) from the arm of the patient at her residence in the neighborhood of Felipe Angeles, Ciudad Juarez, Chihuahua, Mexico (Figure 5). The blood sample was centrifuged at 3000× *g* for 10 min, and the plasma was stored in aliquots of 0.5 mL at −80 °C until tested for arboviruses in C6/36 and Vero-76 cells. A convalescent blood sample was not available for antibody testing. In addition, the female patient did not report any travel history prior to the onset of symptoms. The deidentified medical history and deidentified blood sample were provided by one of the authors (A.M.C.) to another author (D.M.W.) at the University of Texas at El Paso (UTEP), Texas, to test for arboviruses. The patient was enrolled in accordance with the Ethics Committee at the Institute of Biomedical Sciences of the Universidad Autonoma de Ciudad Juarez, Mexico, using a written informed consent form and a questionnaire to obtain demographic and clinical information.

### 4.2. Viral Isolation

The plasma sample was diluted 1:10 in cell culture maintenance medium (Minimum Essential Medium (MEM) supplemented with 2% fetal bovine serum (FBS), 1% penicillin–streptomycin, and 1% non-essential amino acids). Two hundred microliters of the diluted plasma sample was inoculated onto confluent monolayers of Vero-76 and C6/36 cells propagated in a T-25 cm^2^ flask and incubated for 1 h at 37 °C and 28 °C in the presence of 5% CO_2_, respectively. Then, 5 mL of the maintenance medium was added to each culture, and the cells and inoculum were incubated for 7 days at 37 °C and 28 °C in 5% CO_2_. Confluent monolayers of Vero-76 and C6/36 cells were inoculated with maintenance medium to serve as controls. The cells were observed for evidence of viral cytopathic effect (CPE) once daily through an inverted microscope. Then, 7 days post inoculation, the Vero and C6/36 cells were scraped off the flasks and the suspensions were clarified by centrifugation at 3000× *g* for 10 min at 4 °C. The clarified cells and supernatants were stored at −80 °C and labeled as passage 1 (p-1). The cell pellets were resuspended in phosphate-buffered saline (PBS) 1×, and aliquots of 20 µL were spotted onto the ringed areas of slides and dried and fixed in cold acetone at −20 °C for 10 min, before storing at −20 °C for subsequent testing of the cell pellets for selected arboviruses using an immunofluorescence assay.

### 4.3. Immunofluorescence Assay (IFA)

The IFA was performed on the cell pellet fixed to the ringed area of the slides using polyclonal antibodies for West Nile, Saint Louis encephalitis, DENV serotypes 1 and 2 (DENV-1, DENV-2), Chikungunya, Western equine encephalitis, and La Crosse. Furthermore, DENV-1 monoclonal antibody (15F3) ascites were included in the test. Briefly, 10 µL of polyclonal or monoclonal antibodies were added to the cell pellets of the slide and incubated at 37 °C for 1 h. Then, the slides were washed twice in PBS 1× and dried, before adding 10 µL of goat anti-mouse IgG antibody conjugated with fluorescein isothiocyanate (1:100 diluted in PBS 1×) and incubating at 37 °C for 1 h. DAPI (4′,6-diamidino-2-phenylindole) was used as a counterstain (1:1000 dilution). The slides were washed twice in PBS 1× and dried, before adding a drop of a glycerol solution (nine parts glycerol + one part PBS) onto the ringed area of the slides. Slides were examined for fluorescence using a 20× objective on a fluorescent microscope (Nikon, Ti-S). Slide controls containing known infected cells with each of the test viruses and/or uninfected cells were subjected to the same procedure. A *Flavivirus* was detected by IFA using *Flavivirus* polyclonal antibodies and shown to be DENV-1 using DENV-1 monoclonal antibody.

### 4.4. Viremia Levels

Tenfold dilutions of the patient’s plasma sample were prepared in maintenance medium, and 50 µL of each dilution was inoculated into a suspension of baby hamster kidney cells (BHK-21 clone 15) propagated in 24-well plates. A plaque assay was then performed to determine the titer of the DENV-1 isolate as described previously [25].

### 4.5. Molecular Identification

RNA was extracted from the Vero-76 and C6/36 cells infected with DENV-1 (p-1) using the QIAamp viral RNA kit (Qiagen, Valencia, CA, USA), following the manufacturer’s protocol. A generic reverse-transcription polymerase chain reaction (RT-PCR) assay was performed to detect nucleic acid from *Flaviviruses*, as previously described [26]. The amplicons obtained were purified and sequenced using the BigDye^®^ Terminator 3.1 Cycle Sequencing Kit (Applied Biosystems, Foster City, CA, USA), with the sequencer 3730xl DNA analyzer (Applied Biosystems). Sequences were edited with the Sequencher 4.6 software (Gene Codes Corporation, Ann Arbor, MI, USA) and compared to other viral sequences deposited in the GenBank database using the BLASTn analysis.

### 4.6. Envelope Gene Sequencing

The pre-membrane (preM) and envelope (E) genes of the DENV-1 isolate (p1) were amplified by RT-PCR [27,28]. Phylogenetic analysis of the E gene of DENV-1 was performed using neighbor joining and maximum probability analysis implemented in MEGA7 [29]. The best-fit model (GTR+ I + G) was determined by the Mr. Model test software. Clades were evaluated by bootstrap analyses with 1000 replicates for maximum probability analyses. Initial trees for the heuristic search were obtained automatically by applying the Neighbor-Join and BioNJ algorithms to a matrix of pairwise distances estimated using the maximum composite likelihood (MCL) approach and then selecting the topology with the superior log-likelihood value.

### 4.7. Viral Whole-Genome Sequencing

DENV-1 RNA was prepared using the NEBNext Ultra II RNA Library Prep Kit (New England BioLabs, Ipswich, MA). Finished libraries were quality-checked on an Agilent Bioanalyzer (Agilent, Santa Clara, CA, USA) and quantified by real-time PCR. Libraries were pooled and sequenced on a NextSeq 550 (Illumina, San Diego, CA, USA) using the High-output kit (Illumina, San Diego, CA, USA) and a paired-end 75 base read protocol. Raw reads were filtered to remove low-quality reads and adapter sequences using the Trimmomatic package [30]. The read assembly program ABySS was used to assemble the filtered reads into longer de novo contigs to assemble the genome. The genome sequence of DENV-1 was submitted to GenBank with the accession number MZ3432597.

Sixty-eight DENV-1 sequences, as well as DENV-2 New Guinea C to root the tree, were downloaded from GenBank (Table S1). The resulting set of 69 strains from GenBank plus the 2015 isolate from Ciudad Juarez, Mexico, were aligned using Clustal Omega in MegAlign Pro version 17.0.0 (DNASTAR Inc., Madison, WI, USA) and trimmed to remove the 5′ and 3′ UTRs. Maximum-likelihood analysis was performed using the phangorn package [31] in R version 3.5.3 (R Core Team 2017). The GTR + G + I model of nucleotide substitution was selected from the 24 options analyzed as part of the model test analysis in phangorn on the basis of minimizing the AIC score. A maximum likelihood tree was generated using a rooted UPGMA tree as a starting point, and bootstrapping was performed with 1000 iterations. The resulting tree was visualized using iTOL version 5.7 [32].

### 4.8. Replication Curve

The infectivity replication curve of the DENV-1 isolate was determined in Vero-76 and C6/36 cells by plaque assay in BHK-21 clone 15 cells [6]. The C6/36 cells were grown in a T-75 cm^2^ flask and inoculated with the DENV-1 isolate (p-1 from C6/36 cells supernatant) at a multiplicity of infection (MOI) of 0.01. Cells were harvested once CPE became evident at 7 days post infection (dpi), and aliquots of the clarified supernatant (p-2) were stored at −80 °C until used to determine the virus infectivity replication curves. Briefly, Vero-76 and C6/36 cells were inoculated in triplicate with the DENV-1 suspension (p-2) at an MOI of 0.01 and incubated at 37 °C and 28 °C, respectively, for 1 h. Then, the inoculum was removed, and the cells were washed three times with sterile PBS 1× pH 7.4. Five milliliters of maintenance medium was added to cells of each flask, and a 0.5 mL aliquot was collected immediately (time point 0) and replaced with 0.5 mL of new medium. Cells were incubated as mentioned above, and aliquots were collected and replaced with new medium every day until 10 dpi. Timepoint aliquots were tested to determine virus infectivity titers by plaque assays on BHK-21 clone 15 cells. Statistical differences in the viral replication titers were determined using Student’s *t*-test.

## Figures and Tables

**Table 1:** Amino-acid changes in the Mexican DENV-1 strains in comparison to the DENV-1 Ciudad Juarez strain.

Tree Name	Collection Year	Strain Name	GenBank Accession No.	NS1	NS2A		NS3	
				139	41	218	332	338
DENV1_Mexico_2006_BID_V3658	2006	BID_V3658	GU131958.1	D	L	K	V	R
DENV1_Mexico_2007_BID-V3673	2007	BID-V3673	GU131964.1	D	L	K	V	R
DENV1_Mexico_2007_BID-V3679	2007	BID-V3679	GU131966.1	D	L	K	V	R
DENV1_Mexico_2007_BID-V3709	2007	BID-V3709	GQ868513.1	D	L	K	V	R
DENV1_Mexico_2007_BID-V3727	2007	BID-V3727	GQ868521.1	D	L	K	V	R
DENV1_Mexico_2007_BID-V3739	2007	BID-V3739	GQ868527.1	D	L	K	V	R
DENV1_Mexico_2007_BID-V7572	2007	BID-V7572	KJ189317.1	D	L	K	V	R
DENV1_Mexico_2007_BID-V7589	2007	BID-V7589	KJ189323.1	D	L	K	V	R
DENV1_Mexico_2007_BID-V7590	2007	BID-V7590	KJ189324.1	D	L	K	V	R
DENV1_Mexico_2008_BID-V3758	2008	BID-V3758	GQ868537.1	D	L	K	V	R
DENV1_Mexico_2011_BID-V7298	2011	BID-V7298	KJ189306.1	D	L	K	V	R
DENV1_Mexico_2011_BID-V7624	2011	BID-V7624	KJ189348.1	D	L	K	V	R
DENV1_Mexico_2011_BID-V7625	2011	BID-V7625	KJ189349.1	D	L	K	V	R
DENV1_Mexico_2012_BID-V8195	2012	BID-V8195	KJ189368.1	D	L	K	V	R
**DENV1_Mexico_2015_CiudadJuarez**	2015	CiudadJuarez	MZ343259	E	F	R	A	K

## Data Availability

Data is provided in the supplementary table of the article.

## Supplementary Materials

The following are available online at https://www.mdpi.com/article/10.3390/pathogens10070872/s1: Table S1. DENV strains for phylogenetic analysis. Strain names, accession numbers, and country and year of collection are included.

## References

[1] Kraemer M.U., Sinka M.E., Duda K.A., Mylne A., Shearer F.M., Brady O.J., Messina J.P., Barker C.M., Moore C.G., Carvalho R.G. (2015). The global compendium of Aedes aegypti and Ae. albopictus occurrence. Sci. Data.

[2] Hahn M.B., Eisen L., McAllister J., Savage H.M., Mutebi J.P., Eisen R.J. (2017). Updated Reported Distribution of Aedes (Stegomyia) aegypti and Aedes (Stegomyia) albopictus (Diptera: Culicidae) in the United States, 1995–2016. J. Med. Entomol..

[3] San Martín J.L., Brathwaite O., Zambrano B., Solórzano J.O., Bouckenooghe A., Dayan G.H., Guzmán M.G. (2010). The epidemiology of dengue in the Americas over the last three decades: A worrisome reality. Am. J. Trop. Med. Hyg..

[4] De Lourdes Muñoz M., Mercado-Curiel R.F., Diaz-Badillo A., Pérez Ramirez G., Black W.C. (2013). Gene flow pattern among Aedes aegypti populations in Mexico. J. Am. Mosq Control. Assoc..

[5] De la Mora-Covarrubias A., Jiménez-Vega F., Treviño-Aguilar S.M. (2010). Geospatial distribution and detection of dengue virus in Aedes (Stegomyia) aegypti mosquitos in Ciudad Juárez, Chihuahua, Mexico. Salud Publica Mex..

[6] Palermo P.M., De la Mora-Covarrubias A., Jimenez-Vega F., Watts D.M. (2019). Serological evidence of Dengue and West Nile Virus human infection in Juarez City, Mexico. Vector Borne Zoonotic Dis..

[7] Jarman R.G., Nisalak A., Anderson K.B., Klungthong C., Thaisomboonsuk B., Kaneechit W., Kalayanarooj S., Gibbons R.V. (2011). Factors influencing dengue virus isolation by C6/36 cell culture and mosquito inoculation of nested PCR-positive clinical samples. Am. J. Trop. Med. Hyg..

[8] Arredondo-García J.L., Aguilar-López E.G., Aguilar Lugo-Gerez J., Osnaya-Romero N., Pérez-Guillé G., Medina-Cortina H. (2020). Epidemiological panorama of dengue in Mexico 2000–2019. Rev. Latin Infect. Pediatr..

[9] González-Durán E., Vázquez-Pichardo M., Torres-Flores J.M., Garcés-Ayala F., Méndez-Tenorio A., Curiel-Quesada E., Ortiz-Alcántara J.M., Castelán-Sánchez H.G., Salas-Benito J.S., Torres-Longoria B. (2018). Genotypic variability analysis of DENV-1 in Mexico reveals the presence of a novel Mexican lineage. Arch. Virol..

[10] Villabona-Arenas C.J., Zanotto P.M. (2013). Worldwide spread of Dengue virus type 1. PLoS ONE.

[11] Carvalho S.E., Martin D.P., Oliveira L.M., Ribeiro B.M., Nagata T. (2010). Comparative analysis of American Dengue virus type 1 full-genome sequences. Virus Genes.

[12] Prestwood T.R., Prigozhin D.M., Sharar K.L., Zellweger R.M., Shresta S. (2010). A mouse-passaged dengue virus strain with reduced affinity for heparan sulfate causes severe disease in mice by establishing increased systemic viral loads. J. Virol..

[13] Duarte dos Santos C.N., Frenkiel M.P., Courageot M.P., Rocha C.F., Vazeille-Falcoz M.C., Wien M.W., Rey F.A., Deubel V., Desprès P. (2000). Determinants in the envelope E protein and viral RNA helicase NS3 that influence the induction of apoptosis in response to infection with dengue type 1 virus. Virology.

[14] OhAinle M., Balmaseda A., Macalalad A.R., Tellez Y., Zody M.C., Saborío S., Nuñez A., Lennon N.J., Birren B.W., Gordon A. (2011). Dynamics of dengue disease severity determined by the interplay between viral genetics and serotype-specific immunity. Sci. Transl. Med..

[15] Tang Y., Rodpradit P., Chinnawirotpisan P., Mammen M.P., Li T., Lynch J.A., Putnak R., Zhang C. (2010). Comparative analysis of full-length genomic sequences of 10 dengue serotype 1 viruses associated with different genotypes, epidemics, and disease severity isolated in Thailand over 22 years. Am. J. Trop Med. Hyg..

[16] Direccion General de Epidemiologia Mexico. Panorama Epidemiologico de Dengue 2015. https://www.gob.mx/cms/uploads/attachment/file/45495/Pano_dengue_sem_52_2015.pdf.

[17] Direccion General de Epidemiologia Mexico. Panorama Epidemiologico de Dengue 2016. https://www.gob.mx/cms/uploads/attachment/file/178952/Pano_dengue_sem_52_2016.pdf.

[18] Direccion General de Epidemiologia Mexico. Panorama Epidemiologico de Dengue 2017. https://www.gob.mx/cms/uploads/attachment/file/285237/Pano_dengue_sem_52_2017.pdf.

[19] Direccion General de Epidemiologia Mexico. Panorama Epidemiologico de Dengue 2019. https://www.gob.mx/cms/uploads/attachment/file/524262/Pano_dengue_52_2019.pdf.

[20] Rawlings J.A., Hendricks K.A., Burgess C.R., Campman R.M., Clark G.G., Tabony L.J., Patterson M.A. (1998). Dengue surveillance in Texas, 1995. Am. J. Trop. Med. Hyg..

[21] Ramos M.M., Mohammed H., Zielinski-Gutierrez E., Hayden M.H., Lopez J.L., Fournier M., Trujillo A.R., Burton R., Brunkard J.M., Anaya-Lopez L. (2008). Epidemic dengue and dengue hemorrhagic fever at the Texas-Mexico border: Results of a household-based seroepidemiologic survey, December 2005. Am. J. Trop. Med. Hyg..

[22] Thomas D.L., Santiago G.A., Abeyta R., Hinojosa S., Torres-Velasquez B., Adam J.K., Evert N., Caraballo E., Hunsperger E., Muñoz-Jordán J.L. (2016). Reemergence of dengue in southern Texas, 2013. Emerg. Infect. Dis..

[23] Chen W.J., Chen S.L., Chien L.J., Chen C.C., King C.C., Harn M.R., Hwang K.P., Fang J.H. (1996). Silent transmission of the dengue virus in southern Taiwan. Am. J. Trop. Med. Hyg..

[24] Fredericks A.C., Fernandez-Sesma A. (2014). The burden of dengue and chikungunya worldwide: Implications for the Southern United States and California. Ann. Glob. Health.

[25] Morens D.M., Halstead S.B., Repik P.M., Putvatana R., Raybourne N. (1985). Simplified plaque reduction neutralization assay for dengue viruses by semimicro methods in BHK-21 cells: Comparison of the BHK suspension test with standard plaque reduction neutralization. J. Clin. Microbiol..

[26] Kuno G., Chang G.J., Tsuchiya K.R., Karabatsos N., Cropp C.B. (1998). Phylogeny of the genus Flavivirus. J. Virol..

[27] Díaz F.J., Black W.C., Farfán-Ale J.A., Loroño-Pino M.A., Olson K.E., Beaty B.J. (2006). Dengue virus circulation and evolution in Mexico: A phylogenetic perspective. Arch. Med. Res..

[28] Warrilow D., Northill J.A., Pyke A.T. (2012). Sources of dengue viruses imported into Queensland, australia, 2002–2010. Emerg. Infect. Dis..

[29] Kumar S., Stecher G., Tamura K. (2016). MEGA7: Molecular Evolutionary Genetics Analysis version 7.0 for bigger datasets. Mol. Biol. Evol..

[30] Bolger A.M., Lohse M., Usadel B. (2014). Trimmomatic: A flexible trimmer for Illumina sequence data. Bioinformatics.

[31] Schliep K.P. (2011). phangorn: Phylogenetic analysis in R. Bioinformatics.

[32] Letunic I., Bork P. (2019). Interactive Tree of Life (iTOL) v4: Recent updates and new developments. Nucleic Acids Res..

