# Supplementary material for: Characterization of a Dengue Virus Serotype 1 Isolated from a Patient in Ciudad Juarez, Mexico
Source: Pathogens. 2021 Jul 10;10(7):872. doi: 10.3390/pathogens10070872 (PMC8308707; doi:10.3390/pathogens10070872)
Supplement: Supplementary file 1 [file pathogens-10-00872-s001.zip › pathogens-1269008-supplementary.pdf]

Table S1. DENV strains for phylogenetic analysis. Strain names, accession numbers, and country and year of collection are included.

Tree Name	Collection Year	Collection Location	Strain Name	GenBank Accession
DENV1_Brazil_1990_Den1BR-90	1990	Brazil	Den1BR-90	AF226685.2
DENV1_Brazil_2010_13861-BR-PE-10	2010	Brazil	13861-BR-PE-10	JX669465.1
DENV1_Brazil_2014_SJRP-2271	2014	Brazil	SJRP-2271	KP188568.1
DENV1_China_2015_GZ-11	2015	China	GZ-11	KX225492.1
DENV1_Colombia_1998_BID-V3377	1998	Colombia	BID-V3377	GQ868560.1
DENV1_Colombia_1998_BID-V7290	1998	Colombia	BID-V7290	KJ189302.1
DENV1_Ecuador_2014_TD-00061-P	2014	Ecuador	TD-00061-P	KY474307.1
DENV1_Florida_2010_BOL-KW010	2010	Florida	BOL-KW010	JQ675358.1
DENV1_France_2014_Toulon-CNR-25329	2014	France	Toulon-CNR-25329	MF004384.1
DENV1_FrenchPolynesia_2001_BID-V2939	2001	French Polynesia	BID-V2939	FJ898448.1
DENV1_Gabon_2012_Gabon2012	2012	Gabon	Gabon2012	MG877557.1
DENV1_Hawaii_2001_Haw03663	2001	Hawaii	Haw03663	DQ672564.1
DENV1_India_1971_715393	1971	India	715393	JQ922546.1
DENV1_Japan_1943_Mochizuki	1943	Japan	Mochizuki	AB074760.1
DENV1_Malaysia_1972_P72-1244	1972	Malaysia	P72-1244	EF457905.1
DENV1_Mexico_2006_BID_V3658	2006	Mexico	BID_V3658	GU131958.1
DENV1_Mexico_2007_BID-V3673	2007	Mexico	BID-V3673	GU131964.1
DENV1_Mexico_2007_BID-V3679	2007	Mexico	BID-V3679	GU131966.1
DENV1_Mexico_2007_BID-V3709	2007	Mexico	BID-V3709	GQ868513.1
DENV1_Mexico_2007_BID-V3727	2007	Mexico	BID-V3727	GQ868521.1
DENV1_Mexico_2007_BID-V3739	2007	Mexico	BID-V3739	GQ868527.1
DENV1_Mexico_2007_BID-V7572	2007	Mexico	BID-V7572	KJ189317.1
DENV1_Mexico_2007_BID-V7589	2007	Mexico	BID-V7589	KJ189323.1
DENV1_Mexico_2007_BID-V7590	2007	Mexico	BID-V7590	KJ189324.1
DENV1_Mexico_2008_BID-V3758	2008	Mexico	BID-V3758	GQ868537.1
DENV1_Mexico_2011_BID-V7298	2011	Mexico	BID-V7298	KJ189306.1
DENV1_Mexico_2011_BID-V7624	2011	Mexico	BID-V7624	KJ189348.1
DENV1_Mexico_2011_BID-V7625	2011	Mexico	BID-V7625	KJ189349.1
DENV1_Mexico_2012_BID-V8195	2012	Mexico	BID-V8195	KJ189368.1
**DENV1_Mexico_2015_CiudadJuarez**	**2015**	**Mexico**	**Ciudad Juarez**	**MZ3432597**
DENV1_Nicaragua_2004_BID-V653	2004	Nicaragua	BID-V653	EU596501.1
DENV1_Nicaragua_2004_BID-V664	2004	Nicaragua	BID-V664	GQ199873.1
DENV1_Nicaragua_2005_BID-V1071	2005	Nicaragua	BID-V1071	EU482617.1
DENV1_Nicaragua_2005_BID-V534	2005	Nicaragua	BID-V534	JN819402.1
DENV1_Nicaragua_2005_BID-V536	2005	Nicaragua	BID-V536	FJ850113.1
DENV1_Nicaragua_2005_BID-V606	2005	Nicaragua	BID-V606	EU596504.1
DENV1_Nicaragua_2005_BID-V621	2005	Nicaragua	BID-V621	FJ410290.1

DENV1_Nicaragua_2005_BID-V629	2005	Nicaragua	BID-V629	FJ024485.1
DENV1_Nicaragua_2006_BID-V746	2006	Nicaragua	BID-V746	JN819403.1
DENV1_Nicaragua_2007_BID-V1223	2007	Nicaragua	BID-V1223	FJ898433.1
DENV1_Nicaragua_2008_BID-V2652	2008	Nicaragua	BID-V2652	GQ199859.1
DENV1_Nicaragua_2009_BID-V5504	2009	Nicaragua	BID-V5504	JF937635.1
DENV1_Nicaragua_2011_BID-V7672	2011	Nicaragua	BID-V7672	KF973460.1
DENV1_Nicaragua_2012_BID-V7651	2012	Nicaragua	BID-V7651	KF973456.1
DENV1_Nicaragua_2012_BID-V7655	2012	Nicaragua	BID-V7655	KF973458.1
DENV1_Nicaragua_2012_BID-V7690	2012	Nicaragua	BID-V7690	KF973472.1
DENV1_Nicaragua_2012_BID-V7691	2012	Nicaragua	BID-V7691	KF973473.1
DENV1_Nicaragua_2012_BID-V7692	2012	Nicaragua	BID-V7692	KF973474.1
DENV1_Nicaragua_2012_BID-V7696	2012	Nicaragua	BID-V7696	KF973475.1
DENV1_PuertoRico_1986_BID-V2097	1986	Puerto Rico	BID-V2097	FJ562106.1
DENV1_PuertoRico_1992_BID-V2136	1992	Puerto Rico	BID-V2136	FJ410186.1
DENV1_PuertoRico_1993_BID-V2134	1993	Puerto Rico	BID-V2134	FJ410185.1
DENV1_PuertoRico_1994_BID-V2127	1994	Puerto Rico	BID-V2127	FJ410179.1
DENV1_PuertoRico_1995_BID-V1744	1995	Puerto Rico	BID-V1744	FJ205875.1
DENV1_PuertoRico_1996_BID-V2138	1996	Puerto Rico	BID-V2138	FJ478457.1
DENV1_PuertoRico_2006_BID-V852	2006	Puerto Rico	BID-V852	EU482591.1
DENV1_PuertoRico_2012_BID-V7719	2012	Puerto Rico	BID-V7719	KJ189359.1
DENV1_Thailand_1960_606147	1960	Thailand	606147	JQ922547.1
DENV1_Thailand_1982_ThD1-0081-82	1982	Thailand	ThD1-0081-82	AY732481.1
DENV1_Venezuela_1997_BID-V2162	1997	Venezuela	BID-V2162	FJ639735.1
DENV1_Venezuela_1998_BID-V2168	1998	Venezuela	BID-V2168	FJ639740.1
DENV1_Venezuela_1998_BID-V2169	1998	Venezuela	BID-V2169	FJ639741.1
DENV1_Venezuela_1999_BID-V2171	1999	Venezuela	BID-V2171	FJ639743.1
DENV1_Venezuela_2000_BID-V3549	2000	Venezuela	BID-V3549	GU131833.1
DENV1_Venezuela_2004_BID-V2423	2004	Venezuela	BID-V2423	JN819425.1
DENV1_Venezuela_2006_VE-61006	2006	Venezuela	VE-61006	HQ332182.1
DENV1_Venezuela_2007_BID-V1134	2007	Venezuela	BID-V1134	EU482609.1
DENV1_Venezuela_2007_VE-61081	2007	Venezuela	VE-61081	HQ332183.1
DENV1_Venezuela_2008_BID-V2469	2008	Venezuela	BID-V2469	FJ850104.1
DENV2_PapuaNewGuinea_1944_NewGuineaC	1944	Papua New Guinea	New Guinea C	KM204118.1
